# A Deep Reinforcement Learning Approach to Droplet Routing for Erroneous Digital Microfluidic Biochips †

**DOI:** 10.3390/s23218924

**Published:** 2023-11-02

**Authors:** Tomohisa Kawakami, Chiharu Shiro, Hiroki Nishikawa, Xiangbo Kong, Hiroyuki Tomiyama, Shigeru Yamashita

**Affiliations:** 1 Graduate School of Science and Engineering, Ritsumeikan University, Kusatsu 525-8577, Japan; chiharu.shiro@tomiyama-lab.org (C.S.); ht@fc.ritsumei.ac.jp (H.T.); 2 Graduate School of Information Science and Technology, Osaka University, Osaka 565-0871, Japan; nishikawa.hiroki@ist.osaka-u.ac.jp; 3 Department of Intelligent Robotics, Faculty of Engineering, Toyama Prefectural University, Imizu 939-0398, Japan; kong@pu-toyama.ac.jp; 4 College of Information Science and Engineering, Ritsumeikan University, Kusatsu 525-8577, Japan; ger@cs.ritsumei.ac.jp

**Keywords:** biochips, digital microfluidic biochips, deep reinforcement learning, optimization

## Abstract

Digital microfluidic biochips (DMFBs), which are used in various fields like DNA analysis, clinical diagnosis, and PCR testing, have made biochemical experiments more compact, efficient, and user-friendly than the previous methods. However, their reliability is often compromised by their inability to adapt to all kinds of errors. Errors in biochips can be categorized into two types: known errors, and unknown errors. Known errors are detectable before the start of the routing process using sensors or cameras. Unknown errors, in contrast, only become apparent during the routing process and remain undetected by sensors or cameras, which can unexpectedly stop the routing process and diminish the reliability of biochips. This paper introduces a deep reinforcement learning-based routing algorithm, designed to manage not only known errors but also unknown errors. Our experiments demonstrated that our algorithm outperformed the previous ones in terms of the success rate of the routing, in the scenarios including both known errors and unknown errors. Additionally, our algorithm contributed to detecting unknown errors during the routing process, identifying the most efficient routing path with a high probability.

## 1. Introduction

### 1.1. Digital Microfluidic Biochips (DMFBs)

**Figure 1:**
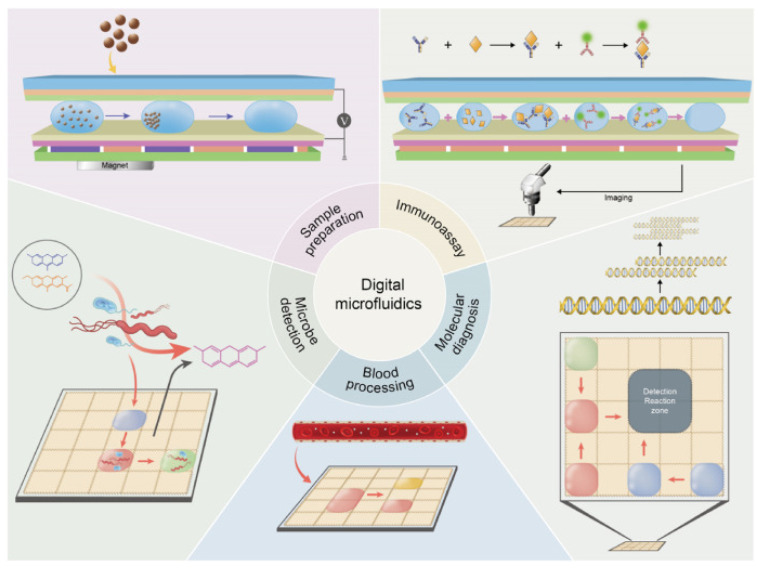
Overview of DMF-based biomedical applications [6].

Digital microfluidic biochips (DMFBs), a subtype of biochips, have transformed the biochemical process industry through their capability to automatically execute operations at a miniature scale. These diminutive, efficient, and user-friendly devices, often referred to as a “lab-on-chip”, mark a significant improvement over the traditional methodologies [1,2]. DMFBs serve a broad spectrum of applications, including DNA analysis, clinical diagnosis, and polymerase chain reaction (PCR) testing [3,4,5]. Figure 1 provides an overview of the wide range of DMF-based biomedical applications in the past decade. It illustrates the progression made in DMF-based immunoassays, molecular diagnosis, blood processing, and microbe detection on automated DMF platforms [6]. Additionally, the COVID-19 pandemic propelled DMFBs into the spotlight, due to their ability to deliver rapid and reliable diagnostic results. For instance, the National Institutes of Health (NIH), a leading U.S. medical research agency, instituted the Rapid Acceleration of Diagnostics (RADx) initiative. RADx’s primary objective was to expedite the development, validation, and commercialization of innovative diagnostic technologies for COVID-19, with biochip technologies playing a pivotal role [7].

From this aspect of research, not only error defections methods and droplet routing algorithms, but also various other kinds of research are developing.For instance, biochip fabrication methodologies are constantly advancing [8], and their integration with emergent technologies such as 5G communication, the Internet-of-medical-things (IoMT), artificial intelligence (AI), and cloud computing is being explored [9]. This convergence is aiding progress towards developing the concept of a hospital-on-chip (HOC).

**Figure 2:**
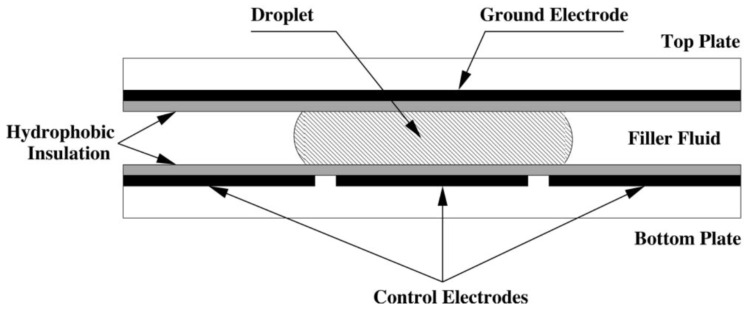
Cross-sectional view of a DMFB [12].

As various types of biochips are being developed, DMFBs distinguish themselves from their predecessors by allowing the manipulation of minuscule discrete droplets on a device [10,11,12]. They have the capability to generate droplets from a reservoir, split droplets, mix different droplets, and move multiple droplets simultaneously [13]. As illustrated in Figure 2, a droplet and hydrophobic insulation are placed between a ground electrode and a set of controllable electrodes. By varying the voltage across these controllable electrodes, DMFBs can manipulate droplets and perform a variety of movements. The electrowetting-on-dielectric (EWOD) technique, which manipulates the interfacial tension between a conductive fluid and a solid electrode via an applied electric field, is fundamental to the operation of DMFBs [14].

However, despite these advancements, DMFB reliability is still a significant concern. Previous research has identified several potential error sources within DMFBs, classifiable as known or unknown errors [15,16,17]. Known errors, such as cell degradation, droplet residues due to an unexpected surface tension, and obstacles impeding droplet movement, can be identified prior to the routing process, often being detected by sensors or cameras. While previous droplet routing algorithms have addressed these errors [18,19], unknown errors such as electrode breakdown, unexpected electrode shorts with neighboring electrodes, or fluctuations in temperature and heat that are undetectable by human or mechanical observers are more elusive and challenging.

**Figure 3:**
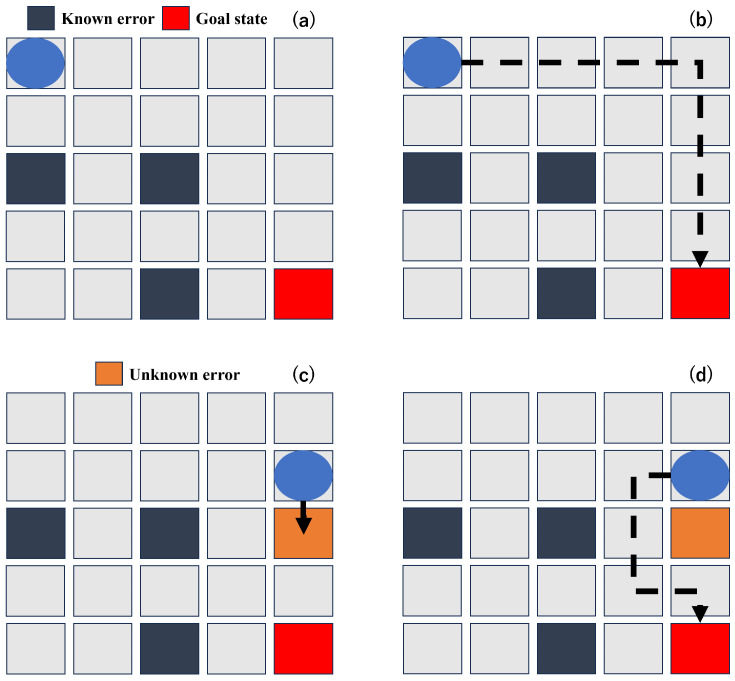
Difference between the previous methods and our method in a situation with unknown errors. (**a**) An initial DMFB environment. (**b**) A routing path of the previous methods. (**c**) A failure case of the previous methods. (**d**) A success case of our method.

Certain unknown errors only surface during the routing process, and they pose significant challenges, due to their unpredictability and difficulty of detection. Figure 3 illustrates a practical scenario where the conventional methods fall short in identifying and adapting to these unknown errors, which ultimately result in a failed routing. The traditional methods rely heavily on data provided by sensors or cameras prior to the routing, limiting their error detection capabilities to known issues like those depicted in Figure 3a. They consequently generate a routing path similar to what is shown in Figure 3b. However, when an unknown error is present in the predetermined routing path, droplets become stuck in a single state. They continue to apply voltage to the unidentified erroneous cell, as portrayed in Figure 3c. This issue stems from the fact that these methods establish a routing path before the routing begins, utilizing information from sensors or cameras. Regrettably, unknown errors evade this detection system and only become evident during the routing process.

In actual biochip experiments, it is crucial to accurately adapt not only known errors but also to unknown errors in real time, since one of the most important factors in biochemical experiments is reliability. Considering this issue, we propose an algorithm that considers both known and unknown errors, reflecting a more authentic biochip environment. By not only including known errors but also unknown ones in the training phase, our model can efficiently handle all types of errors, as shown in Figure 3d.

Not considering all kinds of errors may lead to system failures or accidents. A case in point was Illumina’s NeoPrep device. Introduced in 2015, this $40 K instrument utilized DMFBs to automate DNA sequencing sample preparation [20]. Despite its initial success in infectious disease testing and newborn disease detection, Illumina had to discontinue sales of NeoPrep in 2017 due to significant reliability issues, which indicates the importance of error handling in biochip experiments.

### 1.2. Deep Reinforcement Learning (DRL)

Deep reinforcement learning (DRL) is the combination of two methodologies: deep learning (DL) and reinforcement learning (RL). The main purpose of RL is to solve problems, utilizing intelligent agents that perform actions and aim to maximize the total rewards within a given environment [21]. In addition to the RL approach, the development of DL has significantly contributed to solving more complex tasks. For instance, in 2013, a deep Q learning algorithm was proposed as the first DRL algorithm [22]. By adding the two methods of experience replay and epsilon greedy strategies to the DRL algorithm, in 2015, a DRL model outperformed human experts in some Atari games [23], which was a significant milestone in the development of DRL. Furthermore, the same year also witnessed the historical event of AlphaGO, a DRL model, which defeated a professional human Go player with a resounding score of 5-0 [24]. This accomplishment demonstrated the impressive potential of DRL on a global scale, as Go is a game known for its complexity and strategic depth.Today, the capabilities of DRL extend beyond game playing and into various complicated and high-stakes domains. The technology has been demonstrated in StarCraft at the grand master level [25], natural language processing [26], predictions of 3D models of protein structures [27], and so on.

### 1.3. Related Works

Since 2004, the synthesis process of digital microfluidic biochips (DMFBs) has involved several distinct steps. Initially, biologists developed a bioassay protocol [28]. This protocol was subsequently mapped onto designated electrode areas, also known as fluidic modules, which facilitated the execution of fluidic operations [29].

The transportation of droplets from one module to the next, known as droplet routing, is a critical part of the synthesis process and developed over the past few decades. For instance, Huang et al. introduced a fast routing method and performance-driven approach [30]. They defined an entropy-based routing technique for a better routing method but were unable to efficiently manage delayed delivery times.To address blockages in biochips, Keszocze et al. introduced an exact routing method, which guaranteed optimal solutions to routing paths [31]. Additionally, other strategies have been implemented, such as Pan et al. calculating the Manhattan distance between the source and target to reduce pin count [32]. Even though these innovative approaches brought significant improvements, they came with a trade-off, specifically affecting the resource efficiency of the system. Furthermore, many routing methods have been proposed [33,34,35]. These methods, however, are static and ignore the fact that biochips have various kinds of errors, including known errors and unknown errors [15,16,17].

In order to address the degradation issue, which is a type of known error, an adaptive routing algorithm has been proposed [19]. This algorithm’s primary function is to identify the health conditions of the electrodes, thereby facilitating reliable fluidic operations using a deep reinforcement learning (DRL) algorithm. However, this approach exhibits a limitation in its adaptability to unknown errors. This limitation stems from the fact that the method obtains information about each electrode from a charge-coupled device (CCD) camera [36,37] before initiating the routing process. Consequently, this method falls short in handling the unknown errors that may occur during the routing process or errors that remain undetectable by the CCD camera.

Another notable work in error management involved the proposal of the design of fault-tolerant and dynamically reconfigurable microfluidic biochips [38]. The objective of this approach is to dynamically assign specific modules during the routing process, while taking into account the fault tolerance of these modules. This strategy proves effective in detecting, not just known errors, but also unknown errors, while efficiently utilizing module placement. Despite these advantages, the method exhibits limitations in adapting to the comprehensive range of errors. For example, should an electrode fail during the routing process, a droplet will be immobilized, leading to the failure of the routing process. Additionally, the reassignment of modules to other cells reduces the number of cells available for other droplets to use in parallel.

### 1.4. Paper Contributions

This paper is an extended version of [39], offering a more in-depth analysis of the algorithm’s performance and a broader range of experiments. The contributions of this paper are outlined below.

- This paper presents a new deep reinforcement learning-based routing algorithm for digital microfluidic biochips (DMFBs);
- It contributes to the field by addressing the crucial issue of error management in DMFBs, specifically both known and unknown errors. It proposes and tests an algorithm that can effectively handle different types of errors, potentially boosting the reliability and efficiency of biochips;
- In addition to proposing a new algorithm, this paper conducted extensive experiments to compare the performance of this algorithm against existing ones. The comprehensive results demonstrated the superior performance of the proposed algorithm in terms of accuracy, optimality of the routing path, and error detection capability.

In this paper, we first introduce a new deep reinforcement learning-based routing algorithm for digital microfluidic biochips (DMFBs) in Section 2. We then provide a detailed description of the proposed framework in Section 2.1, delving into the particulars of the environment in Section 2.2 and details of the agent in Section 2.3. In the subsequent part of the paper, we focus on the verification of our proposition. Section 3 contains a comprehensive account of the experiments conducted. The experimental setup is detailed in Section 3.1, while Section 3.2 elucidates the process of agent training. Subsequent to these, Section 3.3 presents the results of the experiments. We finally draw the paper to a close in Section 4 with the conclusions, providing a summary of the key research findings and their implications for the relevant field.

## 2. Proposed DRL-Based Routing Algorithm

### 2.1. Framework Description

**Figure 4:**
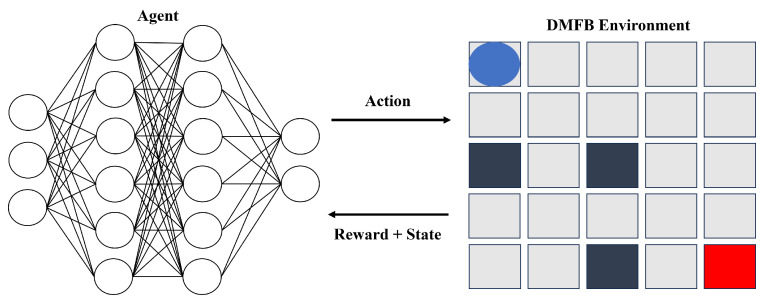
The proposed framework for the agent and DMFB environment interaction. The left image represents the agent, represented by a deep neural network (DNN), while the right image shows the initial state of the DMFB environment.

The framework we propose revolves around the interactive dynamics between an intelligent agent and a digital microfluidic biochip (DMFB) environment. As illustrated in Figure 4, the primary objective of this framework is to maximize the cumulative reward the agent obtains from the DMFB environment.

### 2.2. Environment

An integral part of the proposed framework is the creation of a digital microfluidic biochip (DMFB) environment. Initially, the DMFB environment designates the upper-left state as the starting point and the lower-right state as the goal. It also introduces both known and unknown errors at random positions, to consider real DMFB environments that have all kinds of errors. To ensure navigability, the DMFB environment employs the breadth-first search (BFS) algorithm. If the algorithm is unable to find a route from the start state to the goal state within the initially configured DMFB environment, the environment reinitializes itself. This process is repeated until at least one valid path from the starting state to the goal state is established.

Upon successful initialization, the DMFB environment begins its interaction with the agent. It accepts an action input from the agent and, in response, provides the corresponding reward and state updates. Given the nature of DMFB operations, a droplet has four possible movements at any given moment: up, down, right, and left. Therefore, the DMFB environment also offers these four choices of action. The current state provided by the DMFB environment includes the droplet’s status, the goal state, and the presence of any known errors, which are provided from a CCD camera in real experiments [36,37]. Furthermore, if a droplet exhibits irregular movements, the DMFB environment incorporates unknown error information into the state. This ability to add unknown error information enhances the proposed algorithm’s adaptability to handling unexpected errors. The rewards provided by the DMFB environment to the agent are detailed in Table 1. The maximum number of steps, as mentioned in the table, is calculated as 2 × (w + h), where “w” represents the width and “h” denotes the height of the biochip. This framework allows the algorithm to navigate efficiently in a DMFB environment, making it an integral part of the system’s adaptive learning process.

### 2.3. Agent

In our proposed framework, the agent is represented by a deep neural network (DNN), leveraging a convolutional neural network (CNN) [40] architecture. The fundamental purpose of the learning process is to find the optimal configuration of parameters that best fits the model. This process dynamically interacts with the digital microfluidic biochip (DMFB) environment, determining the agent’s behavior and its learning trajectory.

The CNN agent utilizes a 3D array input, which contains critical information pertaining to the current state of the droplet, the desired goal state, and the presence of any known errors. If we denote the size of the biochip (w × h), the size of the input array is then configured as (w, h, 3). This structure guarantees a comprehensive representation of the DMFB environment, enabling the agent to make informed and effective decisions.

The detailed architecture of the CNN can be seen in Table 2. It follows a layered structure, beginning with convolutional layers, which are primarily tasked with feature extraction. The first layer utilizes 32 filters, while the second and third layers use 64 filters each. All three layers employ the ReLU (rectified linear unit) activation function, introduced to add non-linearity into the model. Following the convolutional layers, two linear layers are deployed. The first possesses 256 nodes and also applies the ReLU activation function. The second linear layer, depending on its function, may have four nodes for the actor network or a single node for the critic network. The Softmax activation function is applied in this layer, enabling it to generate a probability distribution for the agent’s potential actions.

As the agent continually interacts with and adjusts to the DMFB environment, it refines its understanding of the system. This leads to the optimization of its actions, thereby maximizing the cumulative rewards. As a result, the agent’s performance in navigating the DMFB environment improves.

## 3. Simulation Experiments

### 3.1. Simulation Setup

The simulation experiments conducted in this study involve a rigorous exploration of the performance and response of varying sizes of biochip. To ensure a thorough understanding of the system behavior, a total of nine different biochip sizes were evaluated. The specific sizes selected for this study included: 10×10, 10×15, 10×20, 15×15, 15×20, 15×25, 20×20, 20×25, and 25×25. Here, the size refers to the number of cells in the biochips.

In order to better understand the system’s robustness against errors and to simulate real-world conditions, error rates were systematically incorporated in the test cases. These error rates ranged from 0% to 10% for each biochip size. Specific combinations of known and unknown error rates were used, such as (0, 5), (5, 5), and (0, 10). Each tuple represents the known error rate first, followed by the unknown error rate. The simulation experiments were carried out on a machine with the following specifications:

- GPU: GeForce RTX 3060 LHR
- CPU: Core i7-12700F
- RAM: 80 GB

### 3.2. Agent Training

In the training process, we leveraged the proximal policy optimization (PPO) algorithm [41]. The PPO algorithm is an advanced form of policy gradient method that amalgamates principles of the Actor–critic (A2C) method [42]. It is an evolutionary development from the trust region policy optimization (TRPO) algorithm [43] and is favored for its balance between algorithmic complexity and performance outcomes. Our training process is structured into epochs, with each epoch encompassing 10,000 games. Following the conclusion of each epoch, we undertake a comprehensive evaluation of the model against a set of 100 diverse test cases. The model’s performance is considered satisfactory if it can successfully discover a routing path in every single test case.

**Figure 5:**
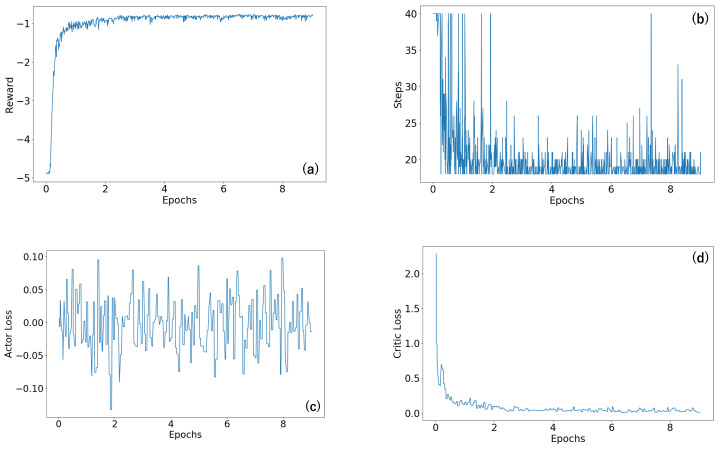
The training trajectory of the size 10×10 biochip. (**a**) The trajectory of the reward value. (**b**) The trajectory of the number of steps. (**c**) The trajectory of the actor loss. (**d**) The trajectory of the critic loss.

**Figure 6:**
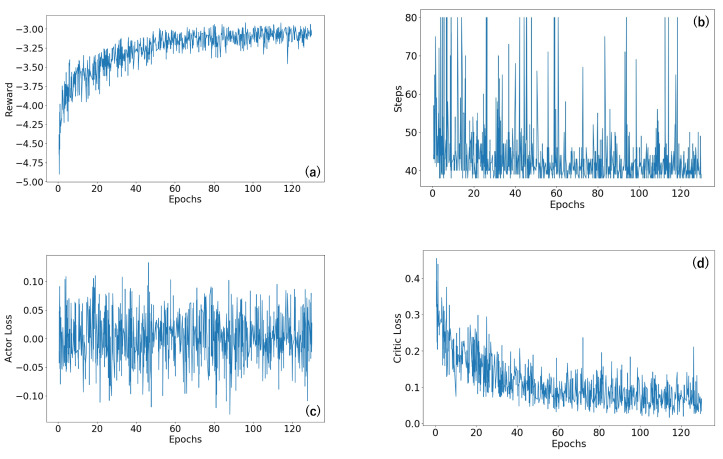
The training trajectory of the size 20×20 biochip. (**a**) The trajectory of the reward value. (**b**) The trajectory of the number of steps. (**c**) The trajectory of the actor loss. (**d**) The trajectory of the critic loss.

In Figure 5 and Figure 6, we provide a thorough depiction of the training trajectories, illustrating the accumulated rewards from the environment, the number of steps taken by the agent throughout the training process, and the loss of each network, actor, and value network. These trajectories are shown for two different biochip sizes: 10×10 and 20×20.

Figure 5 provides a detailed visual breakdown of the trajectories for the 10×10 biochip size, considering an error rate denoted by (5, 5)—representing the rate of known errors and the rate of unknown errors respectively. The agent’s rewards, depicted in Figure 5a, gradually increase, suggesting the agent’s success in refining its policy to maximize rewards from the environment. Concurrently, the number of steps, displayed in Figure 5b, decreases, indicating that the agent learned to find the routing path more efficiently over the training period. The actor loss, exhibited in Figure 5c, corresponds to the loss of the actor network, which is responsible for guiding the agent’s policy. In contrast, the critic loss, displayed in Figure 5d, pertains to the loss of the critic network, which evaluates the agent’s value function. As shown in Figure 5, both the total and critic losses decreased as the training process advanced, while the actor loss hovered around zero. This trend indicated a continuous improvement in the agent’s performance, as it learned to balance exploration and exploitation. Remarkably, in the scenario of a 10×10 biochip size with an error rate denoted by (5, 5)—representing the rate of known errors and the rate of unknown errors, respectively—the agent required only approximately 22 min to satisfactorily accomplish the routing process for all test cases.

In comparison, Figure 6 traces the trajectories for a larger 20×20 biochip size, considering the same error rate denoted by (5, 5). In this case, the agent required 16 h to satisfactorily complete the routing process across all test cases. Despite the difference in training times for the two biochip sizes, our reinforcement learning agent demonstrated promising training in handling both small and larger problem instances.

### 3.3. Simulation Results

In Table 3, we provide data on the performance of the routing algorithms in the test cases. The table presents several simulated results that compared the performance of the proposed routing method versus an existing method [19] across various chip sizes and error rates. The table includes six columns, each representing a different factor. “Chip size” represents the physical dimensions of the chip being tested. “Error rate” is the frequency of known and unknown errors occurring, represented as a pair of rates (known errors, unknown errors). “Routing success” shows the success rate of the droplet in reaching its target; this is divided into the proposed method and the existing one. “Optimal path rate” is the rate at which the droplet uses the shortest routing path, computed using the breadth-first search (BFS) algorithm. “Unknown error detection” measures how many unknown errors were detected during the routing process. We will now dive deeper into interpreting these results and evaluating their implications.

The experimental results showcase a significant comparison between the proposed routing algorithm and the existing methods across different chip sizes and error rates. The results make it evident that the proposed algorithm outperformed the existing methods in almost all aspects and conditions.

**Figure 7:**
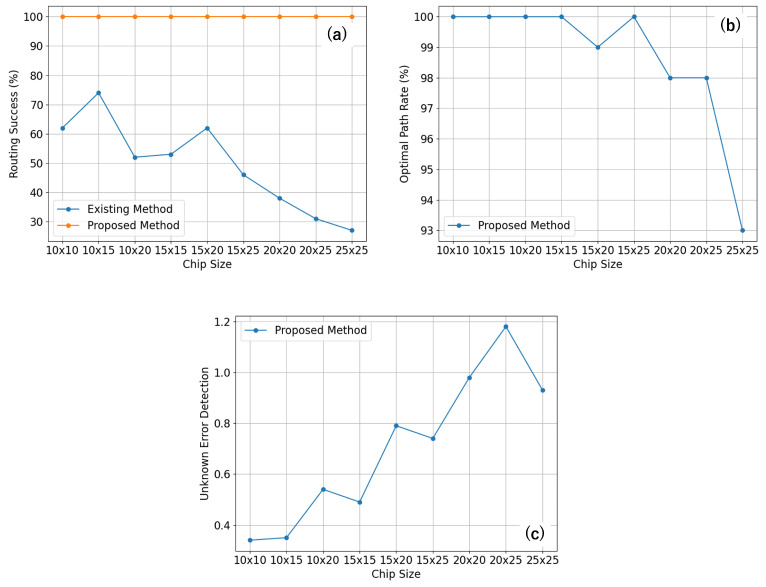
Comparison of experimental results across different chip sizes. (**a**) Routing success for the existing method and the proposed method. (**b**) Optimal path rate for the proposed method. (**c**) Unknown error detection for the proposed method.

Starting with the chip size of 10×10, for all error rates, the proposed algorithm showcased a perfect routing success rate of 100%. This was substantially higher than the routing success rates of the existing methods, which varied from 58% to 62%. Such results illustrate the robustness of the proposed algorithm, even in the presence of errors. This trend was consistent across all chip sizes, where the proposed algorithm maintained a 100% success rate, while the existing methods showed a decreased success rate with larger chip sizes and higher error rates. For instance, for the 20×25 chip size with an error rate of (0, 10), the existing method had a significantly lower success rate of 6%, compared to the 100% success rate of the proposed algorithm. Figure 7a provides a visual comparison of the routing success between the existing method and the proposed method across different chip sizes.

Furthermore, the "optimal path rate" showed how efficiently the routing was performed. A higher rate indicated that the routing was carried out using the minimum number of cells, which signified a more efficient and optimal path. For all chip sizes and error rates, the proposed algorithm consistently outperformed the existing method, often achieving a 100% rate. This signified that, not only was the proposed algorithm more successful in routing, but it also did so in the most optimal way. However, it is important to understand why the optimal path rate is not always 100%. The routing process is a dynamic one, and during this process, if an unknown error is detected in a state near to the droplet, the proposed algorithm smartly avoids this error. This detour results in a path that sometimes deviates from the originally calculated optimal path, which, in turn, lowers the optimal path rate. This mechanism allows for a safer and more reliable routing process, even if this means straying from the optimal path. Figure 7b illustrates the optimal path rate for the proposed method across different chip sizes.

Lastly, “unknown error detection” captures the number of unknown errors detected during the routing process. A higher value is desirable, as this signifies a better error detection capability. This is not just about managing current routing tasks. Detecting unknown errors plays a critical role in facilitating smoother routing in the future. As an error is detected, it becomes a known factor that the routing algorithm can account for in subsequent computations. This enables the algorithm to make more informed routing decisions, effectively avoiding previously detected error zones. It is worth noting that, as the chip size and error rates increased, the proposed algorithm tended to detect more unknown errors, ranging from 0.34 to 2.55. This robust error detection capability, therefore, ensures not just the success of the current routing process, but it also significantly enhances the efficiency and reliability of future routing operations. Figure 7c shows the unknown error detection for the proposed method across the different chip sizes.

Overall, the experimental results provide compelling evidence of the superior performance of the proposed algorithm over existing methods. This superiority was observed across various chip sizes and error rates, highlighting the proposed algorithm’s robustness, efficiency, and improved error detection capabilities. These attributes make it a highly promising alternative for routing in biochips, potentially leading to more accurate results and fewer process interruptions due to errors.

## 4. Conclusions

In conclusion, this research introduced a deep reinforcement learning-based routing algorithm for digital microfluidic biochips (DMFBs). The significance of this work lies in the fact that it not only accounts for known errors but also effectively manages unknown errors, which have been a major reliability issue for biochips.

Our experimental results provide strong evidence of the algorithm’s superior performance in three key areas. First, it demonstrated an impressive success rate in routing, consistently outperforming the previous methods across all tested chip sizes and error rates. Second, the proposed algorithm showed exceptional proficiency in identifying the most efficient routing paths. Even in scenarios where unknown errors were detected during the routing process, the algorithm smartly adapted to the new circumstances, ensuring a successful routing outcome, even if this meant diverging from the initially computed optimal path. Finally, the algorithm’s capability to automatically detect unknown errors during the routing process was a critical asset. This feature did not just enhance the current routing process, it also improved the efficiency and reliability of future routing tasks. By turning unknown errors into known factors, the algorithm evolved to become more informed and adaptive, further strengthening its robustness. In terms of accuracy, the optimality of the routing path, and the number of detected unknown errors during the routing process, the proposed algorithm outperformed the existing ones, thus offering a substantial improvement for DMFB routing.

Given the critical role of DMFBs in various fields, including DNA analysis, clinical diagnosis, and PCR testing, the proposed algorithm’s superior performance and error management capability present a promising advance towards more reliable and efficient biochip operations. As we move forward, we aim to continue refining our algorithm and expanding its application, to further enhance DMFB reliability and efficiency.

## Figures and Tables

**Table 1:** Reward function for the DMFB Environment.

State	Reward
Reach the goal state	0
Reach the maximum step number	−1.0
Any other state	−0.1

**Table 2:** Convolutional Neural Network Structure.

Type	Depth	Activation	Kernel	Padding
Convolution	32	ReLU	3	1
Convolution	64	ReLU	3	1
Convolution	64	ReLU	3	0
Linear	256	ReLU	N/A	N/A
Linear	4 (1) ^a^	Softmax	N/A	N/A

^a^ For the actor network, the depth would be 4 and for the critic network, the depth would be 1.

**Table 3:** Experimental Results.

Chip Size	Error Rate	Existing Method	Proposed Method		
		**Routing Success**	**Routing Success**	**Optimal Path Rate**	**Unknown Error Detection**
10×10	(0, 5)	62	100	100	0.34
	(5, 5)	58	100	99	0.42
	(0, 10)	58	100	99	0.74
10×15	(0, 5)	74	100	100	0.35
	(5, 5)	69	100	100	0.30
	(0, 10)	32	100	99	0.82
10×20	(0, 5)	52	100	100	0.54
	(5, 5)	55	100	93	0.51
	(0, 10)	2	100	94	1.25
15×15	(0, 5)	53	100	100	0.49
	(5, 5)	53	100	98	0.60
	(0, 10)	22	100	94	1.27
15×20	(0, 5)	62	100	99	0.79
	(5, 5)	42	100	97	0.76
	(0, 10)	16	100	95	1.36
15×25	(0, 5)	46	100	100	0.74
	(5, 5)	43	100	93	0.87
	(0, 10)	9	100	95	1.36
20×20	(0, 5)	38	100	98	0.98
	(5, 5)	30	100	91	0.91
	(0, 10)	17	100	89	0.89
20×25	(0, 5)	31	100	98	1.18
	(5, 5)	34	100	91	1.01
	(0, 10)	6	100	92	2.06
25×25	(0, 5)	27	100	93	0.93
	(5, 5)	33	100	90	1.30
	(0, 10)	8	100	84	2.55
